# Supplementary material for: Vitamin K Epoxide Reductase Complex–Protein Disulphide Isomerase Assemblies in the Thiol–Disulphide Exchange Reactions: Portrayal of Precursor-to-Successor Complexes
Source: Int J Mol Sci. 2024 Apr 9;25(8):4135. doi: 10.3390/ijms25084135 (PMC11050172; doi:10.3390/ijms25084135)
Supplement: Supplementary file 1 [file ijms-25-04135-s001.zip › ijms-2928208-supplementary.pdf]

Supplementary Material

VKORC1-PDI assemblies in the thiol-disulphide exchange reaction: Portrayal of Precursor-to-Successor Complexes

Maxim Stolyarchuk[11], Marina Botnari and Luba Tchertanov[1*]

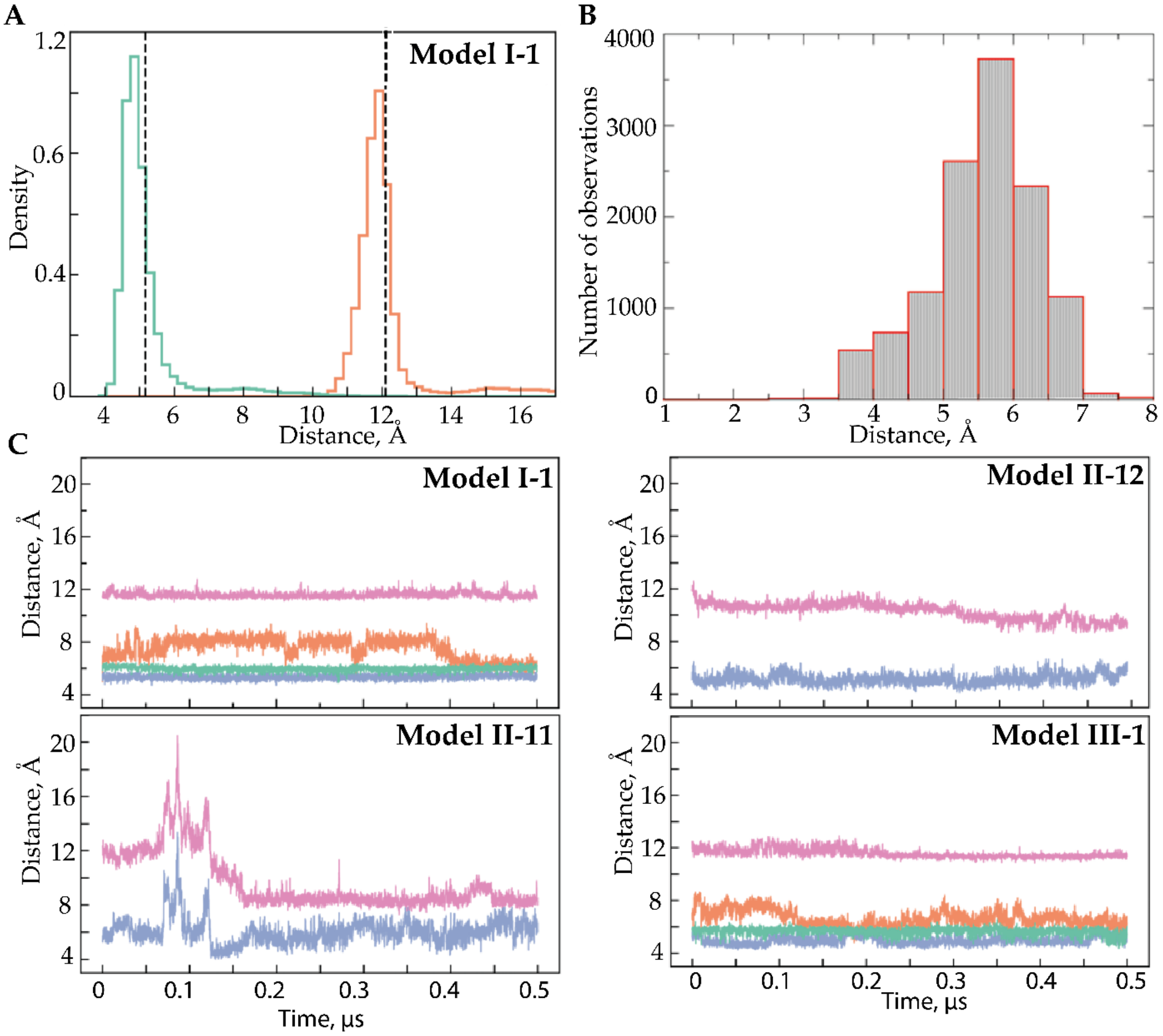

Figure S1. Non-covalent interactions between hVKORC1 and PDI. (**A**) Histogram of the distances between the Cα-atoms of R61 and A42 (orange) and R61 and E46 (cyan) observed during MD simulation of Model I-1. Dashed lines correspond to average distances of 5.2 and 12.1 Å. (**B**) Statistics for the distance between two thiols that are enabled to form a disulphide bond obtained by using protein structures from the Protein Data base (PDB). (**C**) Distance between the Cα-atoms of R61 and A42 (blue), R61 and E46 (pink), C43 and C37 (cyan), and between the sulphur atoms from C43 and C37 (orange) observed during MD simulation of Models I-1, II-11, II-12 and III-1. Residues R61, C43 are from hVKORC1, and A42, E46 and C37 are from PDI.

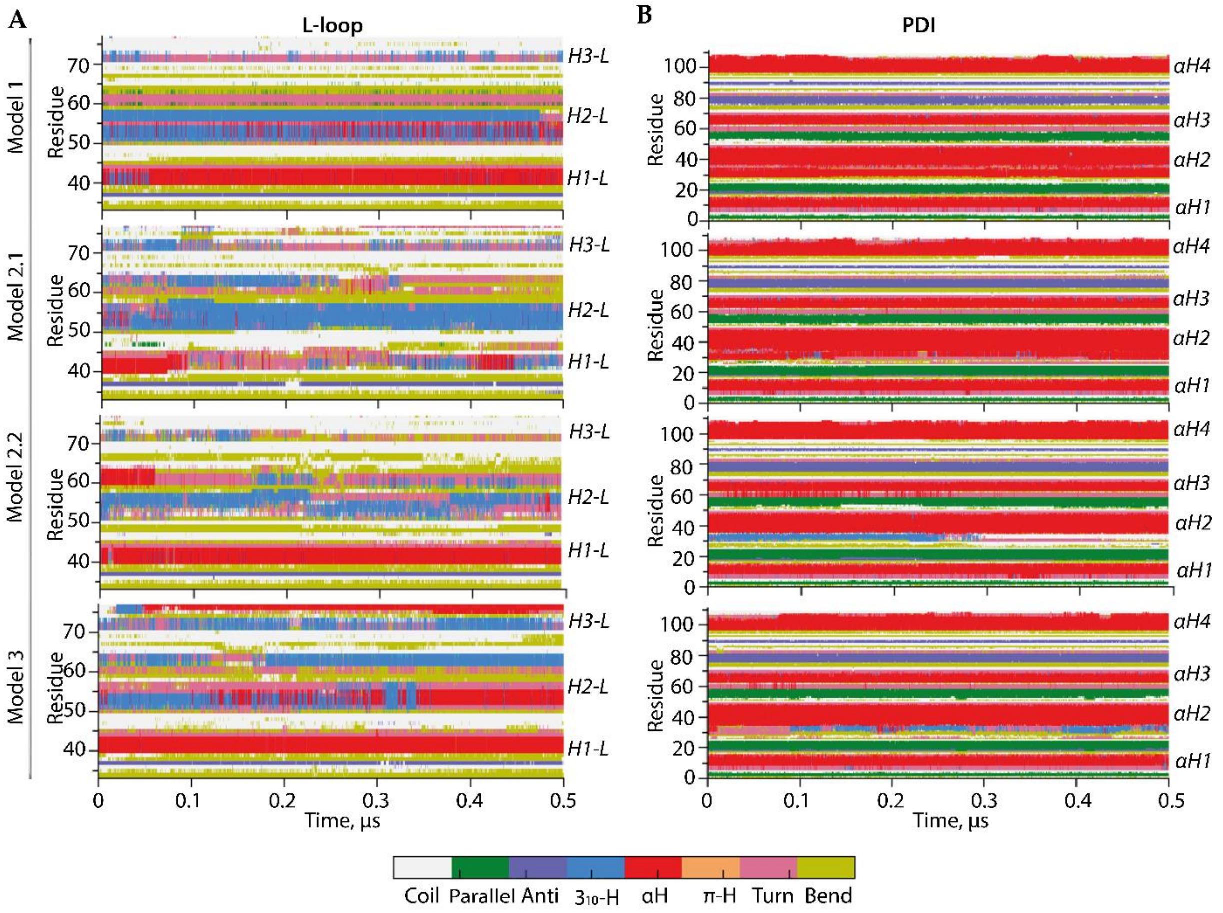

Figure S2. The time-dependent evolution of the secondary structure of each residue in the L-loop (**A**) and PDI (**B**) in PDI-hVKORC1 complexes, represented by Models I, II-1, II-2 and III, as assigned by the Define Secondary Structure of Proteins (DSSP) method: parallel and antiparallel strands are in green and violet; α-, 3_{10}- and π-helices are in red, blue and orange; turn and bend are in pink and sand. The helical structures are enumerated on the right of each figure.

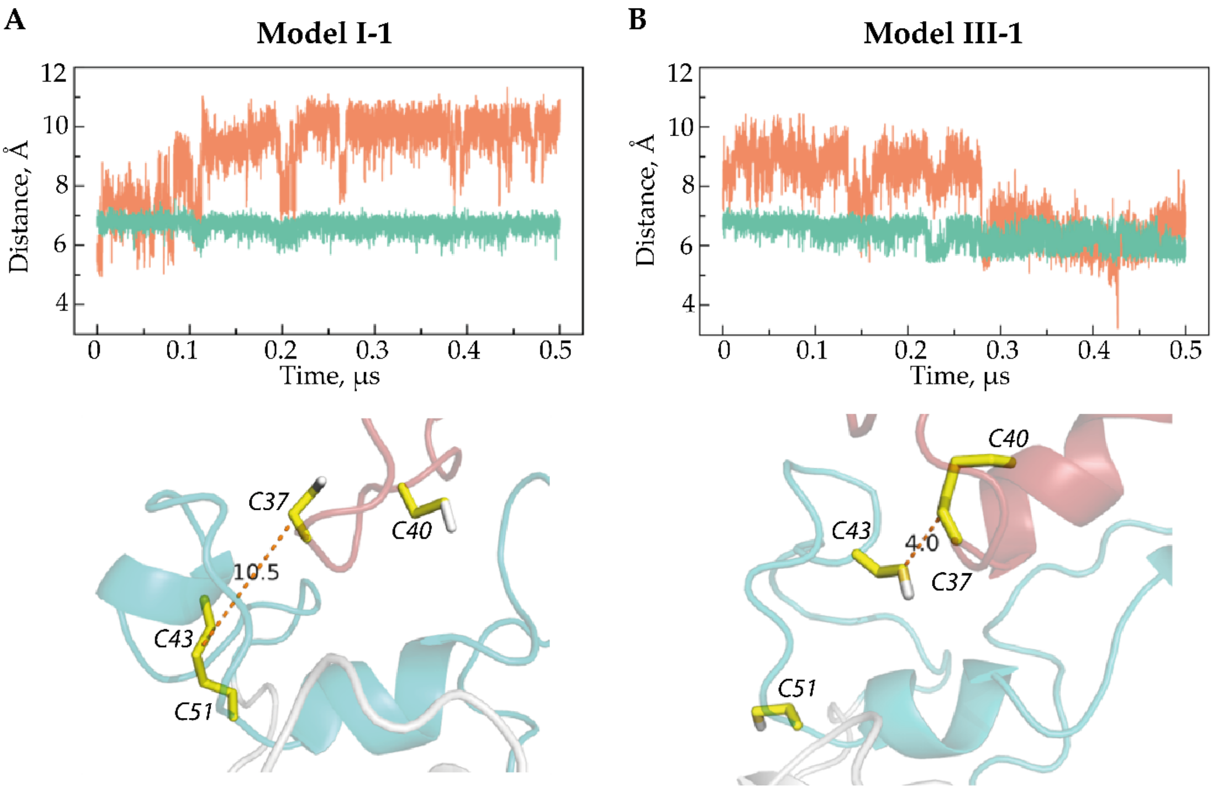

Figure S3. Distances between cysteine residues in hVKOR-PDI 'precursor' (Model I-1) (**A**) and successor' (Model III-1) (**B**) complexes. (Top) Distances between the Cα- and sulphur atoms of C43 and C37 are shown in cyan and orange, respectively. (Bottom) Examples of MD conformations showing atypical distances S···S (dashed lines, values in Å) between cysteine residues. Proteins are shown as cartoons, PDI in red, L-loop in teal; cysteine residues presented as sticks.
